# 

**Published:** 1857-07

**Authors:** 


*Meeting of the American Dental Convention.*—It will be seen by the following note addressed to the publishers of the Journal, that the Convention will meet on Tuesday, the 4th, instead of Tuesday, the 1st of August, as stated in the call published in the last number.—EDS.

CHICAGO, *June* 24, 1857.

GENTLEMEN :

I sent to Dr. Harris the call for the "American Dental Convention," to be published in the American Dental Journal. In the call I said that the Convention would meet in the city of Boston, on Tuesday, the 1st day of August. I should have said, on Tuesday, the 4th day of August, &c.

If not too late, please correct and oblige,

Very truly yours,

W. W. ALLPORT.

